# Discovery of Thieno[3,2‑*b*]pyridine-5-carboxamide and 2,3-Difluorobenzamide Negative Allosteric Modulators of Metabotropic Glutamate Receptor Subtype 5

**DOI:** 10.1021/acsmedchemlett.5c00119

**Published:** 2025-04-22

**Authors:** Katherine E. Crocker, Scott H. Henderson, Rory A. Capstick, David L. Whomble, Aaron M. Bender, Andrew S. Felts, Changho Han, Julie L. Engers, Natasha B. Billard, Mallory A. Maurer, Hyekyung P. Cho, Alice L. Rodriguez, Colleen M. Niswender, Jordan O’Neill, Katherine J. Watson, Sichen Chang, Anna L. Blobaum, Olivier Boutaud, Weimin Peng, Jerri M. Rook, P. Jeffrey Conn, Craig W. Lindsley, Kayla J. Temple

**Affiliations:** † Warren Center for Neuroscience Drug Discovery, 171765 Vanderbilt University, Nashville, Tennessee 37232, United States; ‡ Department of Pharmacology, 12328 Vanderbilt University School of Medicine, Nashville, Tennessee 37232, United States; § Department of Chemistry, Vanderbilt University, Nashville, Tennessee 37232, United States; ∥ Department of Biochemistry, Vanderbilt University, Nashville, Tennessee 37232, United States; ⊥ Vanderbilt Kennedy Center, Vanderbilt University School of Medicine, Nashville, Tennessee 37232, United States; # Vanderbilt Brain Institute, Vanderbilt University School of Medicine, Nashville, Tennessee 37232, United States

**Keywords:** Metabotropic Glutamate Receptor Subtype 5, mGlu_5_, Negative Allosteric Modulator (NAM), Structure Activity Relationship (SAR), Isostere, Levodopa-Induced Dyskinesia, Pain

## Abstract

This Letter describes the discovery of novel mGlu_5_ NAMs **VU6031545** and **VU6024945**. Starting from previously reported picolinamide compounds, a structure–activity relationship study of various core isosteres was conducted, leading to the identification of thieno­[3,2-*b*]­pyridine-5-carboxamide and 2,3-difluorobenzamide as competent core replacements. These compounds are highly potent as well as brain penetrant with an IVIVC agreement and improved oral bioavailability in rats.

## Graphical abstract

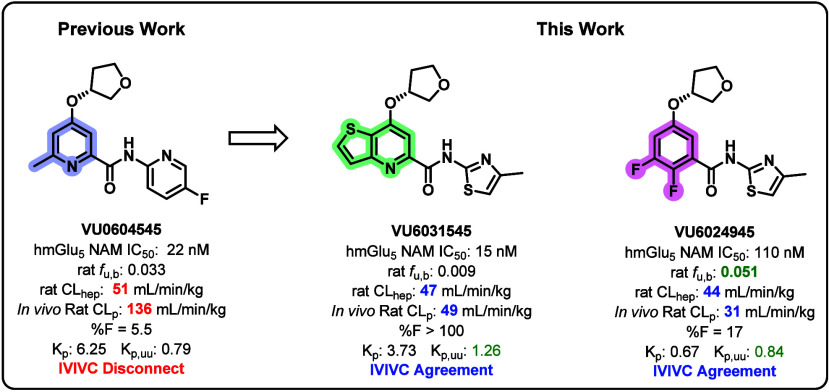

L-Glutamate is the major excitatory neurotransmitter of the mammalian central nervous system (CNS) and modulates the activity of the metabotropic glutamate (mGlu) receptors. The eight mGlu receptor subtypes (mGlu_1–8_) are divided into three groups (groups I, II, and III) based on structure/sequence homology, downstream signaling partners, and pharmacology. Group I metabotropic glutamate receptors (i.e., mGlu_1_ and mGlu_5_) are broadly expressed in the mammalian nervous system and are primarily found post-synaptically where they play a key role in modulating synaptic plasticity.^1^ Predominately coupled via G_q_, activation of mGlu_5_ by glutamate regulates the function of phospholipase C, which, in turn, releases Ca^2+^ from intracellular stores.^2,3^ All eight mGlu receptors have a seven transmembrane (7TM) α-helical domain that connects to a large “Venus fly trap (VFT)” domain. While glutamate binds to the orthosteric site located within the VFT domain, allosteric sites have been identified within the transmembrane domain.^4^ Due to the highly conserved nature of the orthosteric binding site, successful design of selective orthosteric ligands has proven difficult. Therefore, research in the field has shifted focus toward allosteric modulation as an approach to improve selectivity when targeting specific mGlu subtypes. With over a decade of research, mGlu_5_ NAMs are some of the most extensively studied and advanced within the realm of mGlu allosteric modulation.^5–7^ As such, several mGlu_5_ NAMs have been evaluated and demonstrated efficacy both preclinically and clinically, further establishing the utility of a selective mGlu_5_ NAM in a multitude of potential therapeutic applications including levodopa-induced dyskinesia (LID) associated with Parkinson’s disease,^8^ fragile X syndrome,^9^ autism spectrum disorder,^10^ gastroesophageal reflux disease (GERD),^11^ substance abuse disorder,^12^ anxiety,^13^ major depressive disorder,^14^ obsessive-compulsive disorder (OCD),^15^ Alzheimer’s disease,^16^ migraine,^17^ and pain.^18^

Early mGlu_5_ NAM tool compounds MPEP (**1**) and MTEP (**2**) share a biaryl/heterobiaryl acetylene motif as the key pharmacophore which was retained throughout subsequent medicinal chemistry campaigns (Figure 1, analogues **3**–**6**, highlighted in blue). It is well-documented that acetylenes are potentially reactive functional groups and can pose metabolic liabilities. For instance, the mGlu_5_ NAM GRN-529 (**6**) demonstrated extensive glutathione conjugation to the alkyne which is believed to have resulted in biliary epithelial hyperplasia in nonhuman primates (NHPs) during an 8-week regulatory toxicology study.^19^ Attempts to develop nonacetylene based NAMs resulted in the discovery of fenobam (**7**) and AZD9272 (**8**) which were both advanced into clinical trials (Figure 2). Unfortunately, the development of psychosis-like symptoms led to the termination of these trials. It is important to note that further studies attributed these side effects to off-target engagement of monoamine oxidase-B (MAO-B)-mediated mechanisms and not mechanisms facilitated by the mGlu_5_ receptor.^20^ At present, TMP-301 (**9**) is the only nonacetylene-based mGlu_5_ NAM undergoing clinical trials (Phase I) for substance abuse disorders.^21^ Interestingly, TMP-301 (**9**) and AZD9272 (**8**) are structurally very similar (highlighted in Figure 2); thus, TMP-301 may also suffer from off-target side effects (MAO-B). Currently, no mGlu_5_ NAM has successfully progressed through clinical trials, highlighting the continued need for structurally diverse mGlu_5_ NAMs.

**Figure 1:**
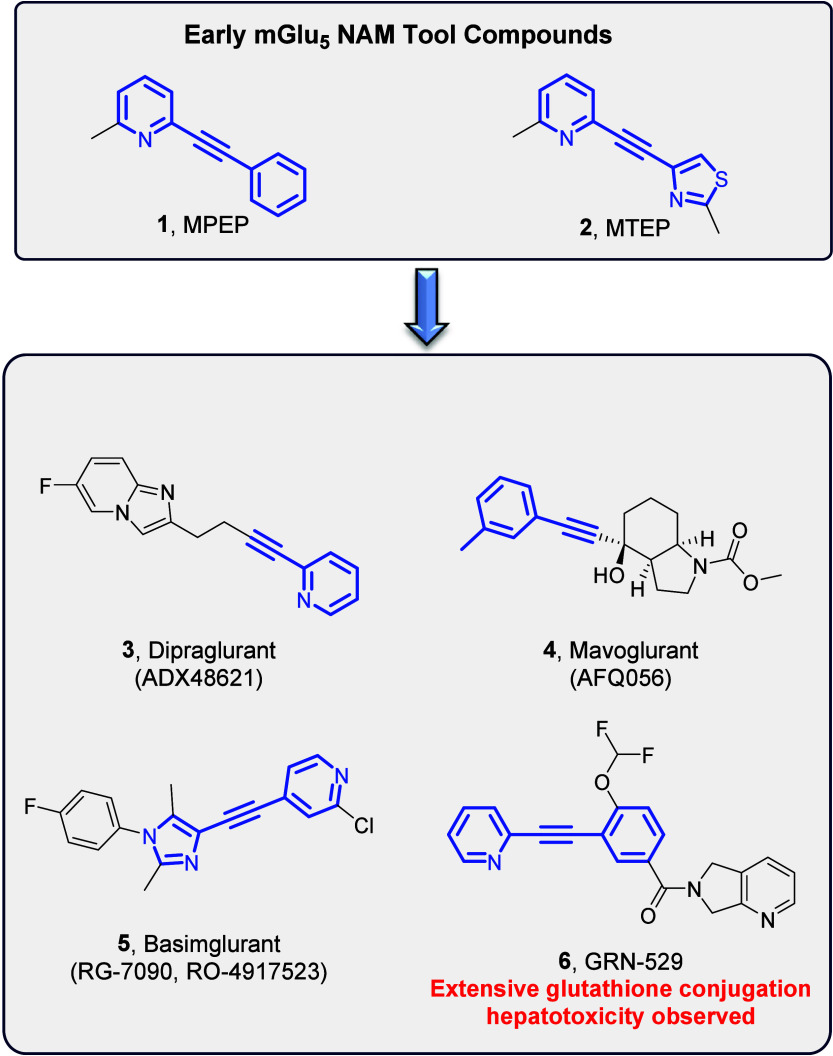
Selective mGlu_5_ NAMs based on an aryl/heterobiaryl acetylene pharmacophore (highlighted in blue).

**Figure 2:**
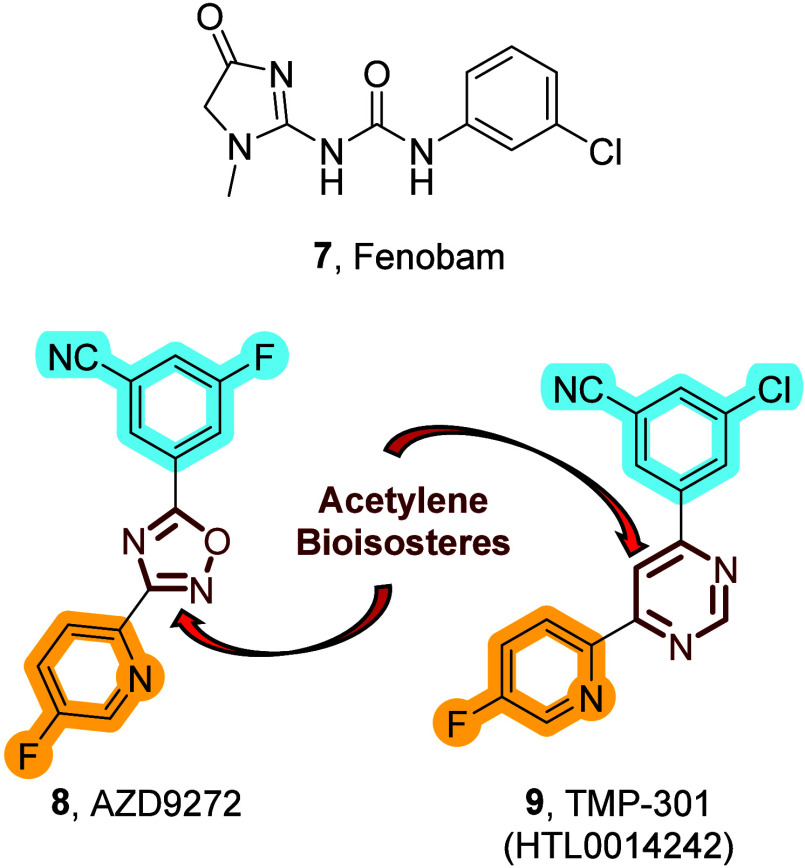
Nonacetylene, selective mGlu_5_ NAMs.

A major focus of our group has been the development of small molecule mGlu_5_ NAMs (Figure 3). Our work led to the identification of preclinical candidate **VU0424238** (**10**) (Figure 3).^22^ In a 28-day toxicology study, a NHP species-specific aldehyde oxidase (AO) metabolite accumulated after 14 days, resulting in pronounced anemia (nonmechanism based); therefore, further development of **10** was halted. Detailed metabolic studies has shown that in NHPs, the pyrimidine headgroup is oxidized by AO, whereas, in rats, this oxidation process is carried out by xanthine oxidase (XO).^23^ Thus, these observed AO/XO metabolism differences between species may be linked to the observed NHP-specific toxicity.

**Figure 3:**
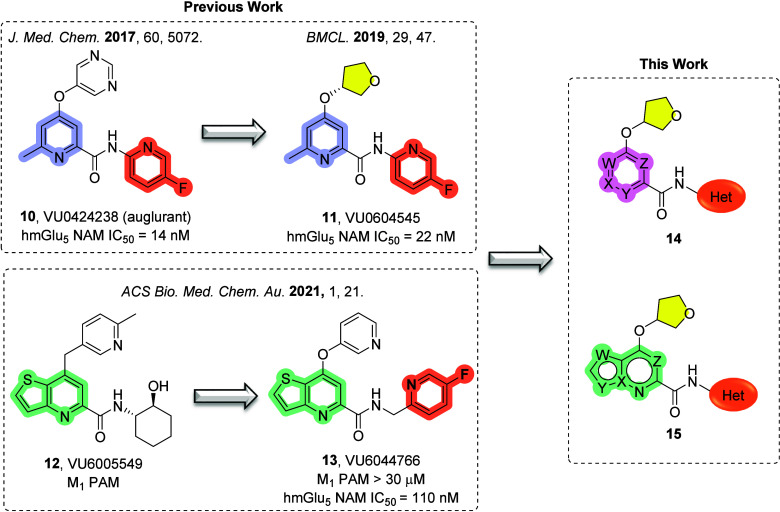
Previously published scaffold-hopping exercises that led to the discovery of mGlu_5_ NAMs **11** and **13**. Further medicinal chemistry efforts led to novel and potent mGlu_5_ NAMs **14** and **15**.

To eliminate AO/XO metabolism, we have published a follow-up series of compounds wherein the pyrimidine headgroup is replaced with a sp^3^-hybridized headgroup as in **VU0604545**, **11** (Figure 3, highlighted in yellow).^24^ Although potent when screened on human mGlu_5_ (hmGlu_5_ IC_50_ = 22 nM), compound **11** suffered several setbacks including high predicted hepatic clearance in rat (CL_hep_ = 51 mL/min/kg), as well as poor oral bioavailability (%F = 5.5). When assessed *in vivo*, clearance was determined to be 136 mL/min/kg, indicating an *in vitro–in vivo* correlation (IVIVC) disconnect. Alternatively, we have reported on a series of 7-alkoxy-thieno­[3,2-*b*]­pyridine-5-carboxamides derived from an unexpected mode shift of an M_1_ PAM scaffold **12** to generate mGlu_5_ NAM **13** (Figure 3).^25^ Compound **13** showed promising mGlu_5_ potency (hmGlu_5_ IC_50_ = 110 nM), was centrally penetrant (*K*_p_ = 0.94) and fully displaced radioligand [^3^H]­methoxyPEPy (*K_i_* = 0.16 μM). Having discovered thieno­[3,2-*b*]­pyridine (Figure 3, green) as a competent core replacement, we performed a scaffold-hopping exercise to incorporate novel cores into our previously established sp^3^-hybridized headgroup series. This endeavor resulted in potent mGlu_5_ NAMs with increased sp^3^ character that lack the metabolically labile pyrimidine found in compound **10** (Figure 3, analogues **14** and **15**).

To begin, we first synthesized 7-(tetrahydrofuran-3-yl)­thieno­[3,2-*b*]­pyridine-5-carboxamides with varying amide moieties (Figure 3, orange) as shown in Scheme 1 (Route 1). Reaction of nitrile **16a** with (*R*)-tetrahydrofuran-3-ol and base afforded nitrile **17a**, which was subsequently hydrolyzed to intermediate carboxylic acid **18a** using sodium hydroxide. Conversion to the acid chloride using phosphorus oxychloride and *in-situ* trapping with various heterocyclic amines generated analogues **19a**, which were screened against human mGlu_5_ to determine potency, with the results highlighted in Table 1. Gratifyingly, the replacement of the picolinamide core with thieno­[3,2-*b*]­pyridine in the context of the (*R*)-tetrahydrofuranyl ether and 5-fluoropyridyl amide led to a compound with similar potency as **11** (**19aB**: hmGlu_5_ IC_50_ = 61 nM). Interestingly, moving the fluoro substituent from the 5-position to the 6-position of the pyridine amide led to a >17-fold reduction in potency (**19aC**: hmGlu_5_ IC_50_ = 1.1 μM); however, substitution with a methyl group at the 6-position resulted in a 3-fold improvement in potency (**19aD**: hmGlu_5_ IC_50_ = 22 nM). Exchanging the pyridine ring of **19aD** with a phenyl ring gave a 40-fold loss in activity (**19aE**: hmGlu_5_ IC_50_ = 845 nM). Exchanging the pyridine ring with a pyrazine was even more detrimental to potency (**19aF**: hmGlu_5_ IC_50_ > 10 μM). Replacement of the 5-methylpyridine with a 4-methylthiazole, a common isostere, provided an analog with similar potency (**19aA**: hmGlu_5_ IC_50_ = 15 nM). Steric bulk on the thiazole amide was not well-tolerated (**19aH**–**19aJ**), nor was the removal of the methyl substituent (**19aK**). Incorporation of fluorine(s) onto the 4-methylthiazole ring resulted in a slight drop in potency (**19aL**: hmGlu_5_ IC_50_ = 55 nM; **19aM**: hmGlu_5_ IC_50_ = 320 nM). Similarly, the addition of an electron-withdrawing group also led to a loss of activity (**19aN:** hmGlu_5_ IC_50_ = 559 nM). Moreover, alternative 5-membered heterocyclic amides were not tolerated (**19aP-R**). Taken together with our previous works, amide preference varied, depending upon the core scaffold employed with no obvious SAR trends identified.

**Scheme 1:**
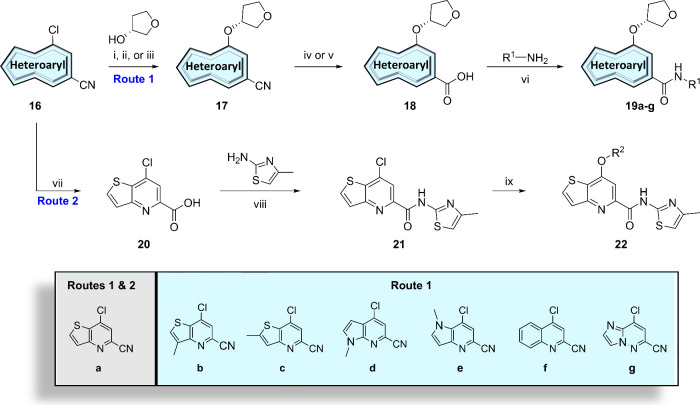
Synthesis of mGlu_5_ NAM Analogues **19a**–**19g** and **22** ^a^

a Reagents and conditions: (i) KHMDS, DMF, 0 °C 1–18 h, 44%–92%; (ii) KHMDS, DMF, 0–60 °C, 48 h, 63%–91% (for **19d**); (iii) K_2_CO_3_, DMF, microwave irradiation at 150 °C, 20 min, 61% (for **19g**); (iv) 2*M* NaOH_(aq)_ or 2*M* LiOH_(aq)_, 1,4-dioxane or THF, 60–100 °C, 2–8 h, 51%–100%; (v) 2*M* NaOH_(aq)_, 1,4-dioxane, microwave irradiation at 120 °C, 30 min, 99%; (vi) POCl_3_, R^1^NH_2_, pyridine, 23 °C, 30 min, 15%–95%; (vii) NaOH_(aq)_, 1,4-dioxane, 100 °C, 18 h, 99%; (viii) POCl_3_, pyridine, 23 °C, 30 min, 74%; (ix) R^2^OH, KO^*t*^Bu, DMSO, 65 °C, 19%–97%.

**Table 1:**
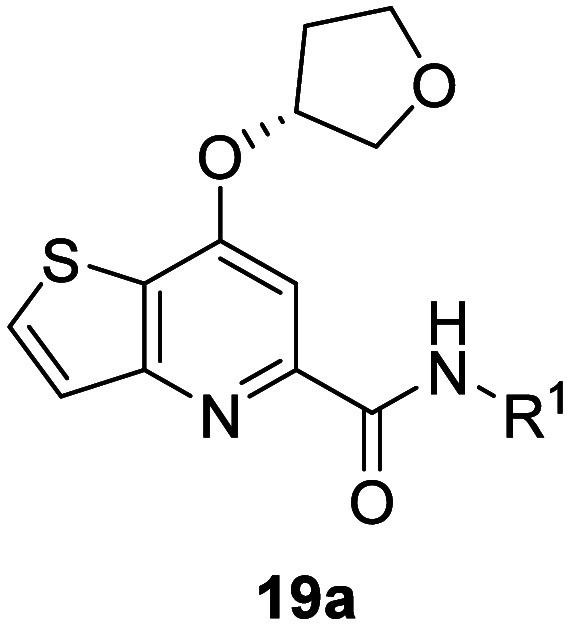

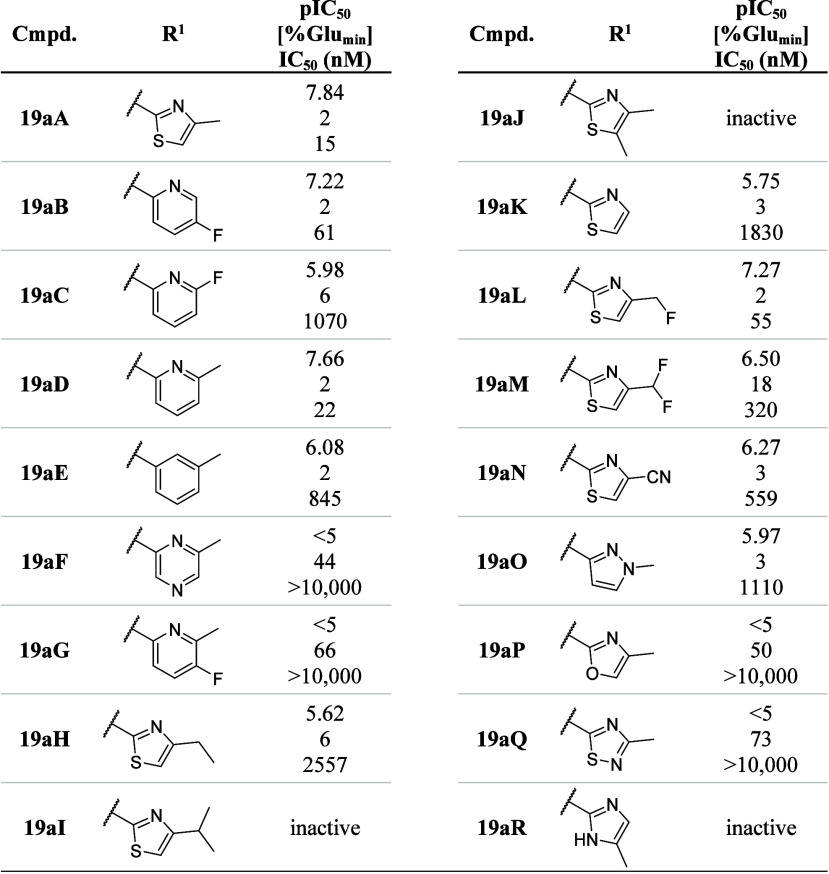
Structures and Activities for Analogs **19aA-aR** ^a^

a Calcium mobilization assays in human mGlu_5_-HEK293A cells were performed in the presence of an EC_80_ fixed concentration of glutamate, *n* = 2 independent experiments in triplicate. The % Glu_Min_ is the measure of efficacy of the NAM to reduce an EC_80_ response of glutamate.

Next, we shifted our attention to the optimization of the headgroup (Figure 3, yellow) in the context of the 4-methylthiazole amide. To generate these analogues, as shown in Scheme 1 (Route 2), nitrile **16a** was first hydrolyzed to the corresponding carboxylic acid **20**. Following acid chloride formation with phosphorus oxychloride, *in-situ* coupling with 4-methylthiazol-2-amine gave rise to the key intermediate **21**. Finally, chloride **21** underwent nucleophilic aromatic substitution with various alcohols to generate compounds **22**, which were screened against human mGlu_5_ with results reported in Table 2. These results highlight the importance of the ether-containing headgroup. When compared to the picolinamide series **11**, several similar SAR trends were noted. For instance, the (*R*)-tetrahydrofuranyl enantiomer (**19aA**: hmGlu_5_ IC_50_ = 15 nM) is preferred to the (*S*)-enantiomer (**22a**: hmGlu_5_ IC_50_ = 543 nM), with a 36-fold difference in potency. Additionally, steric bulk (**22b**) and ring expansion (**22c** and **22d**) were not tolerated and detrimental to potency. Another similarity was observed when ring contraction to the oxetane resulted in a moderately potent compound (**22e**: hmGlu_5_ IC_50_ = 185 nM), a 12-fold loss in activity was observed when compared to **19aA**. Likewise, both the cyclobutanecarbonitrile (**22i**) and thietane (**22j**) analogues also gave an ∼7-fold loss in potency. Like the picolinamide series, homologation to the methylene tetrahydrofuranyl analogue led to an 18-fold loss in potency (**22f**: hmGlu_5_ IC_50_ = 267 nM). Also, conversion from furanyl (**19aA**) to cyclopentyl resulted in a drastic loss of potency (**22h**: hmGlu_5_ IC_50_ > 10 μM), emphasizing the importance of the heteroatom in the ether headgroup.

**Table 2:**
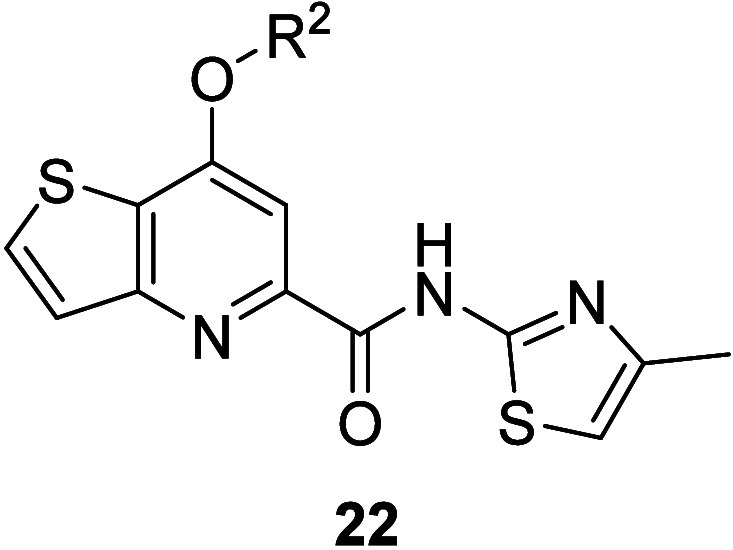

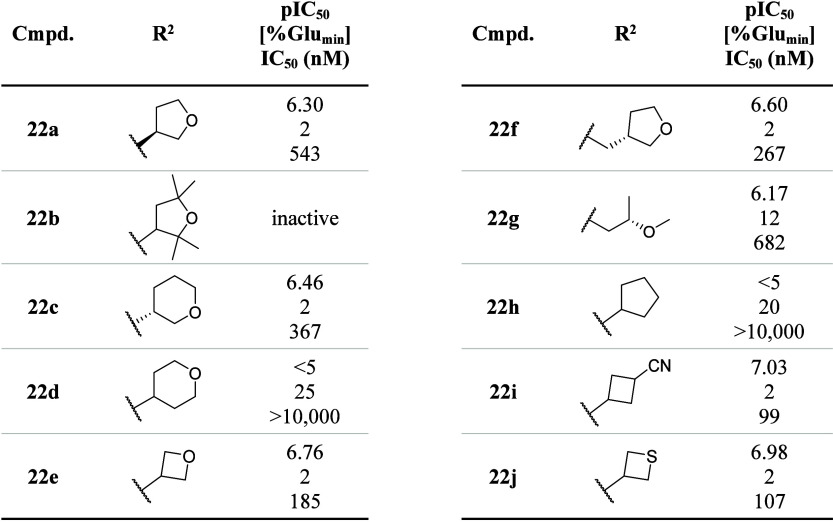
Structures and Activities for Analogues **22a**–**22j** ^a^

a Calcium mobilization assays in human mGlu_5_-HEK293A cells were performed in the presence of an EC_80_ fixed concentration of glutamate, *n* = 2 independent experiments in triplicate. The % Glu_Min_ is the measure of efficacy of the NAM to reduce an EC_80_ response of glutamate.

We next investigated other core replacements of the picolinamide of **11** (Figure 3, blue) while holding constant the (*R*)-tetrahydrofuranyl ether and both the methyl-thiazoleamide and 5-fluoropyridyl amide tails. Synthesis of these analogues followed a similar strategy from easily accessible starting materials, as shown in Schemes 1 and 2. First, various nitriles **16b**–**16g** underwent a series of reactions as previously described for the thieno­[3,2-*b*]­pyridine core analogues to afford compounds **19b**–**19g** (Scheme 1, Route 1). The remaining analogues were synthesized through various ester intermediates that could be easily obtained and subsequently hydrolyzed before performing an amide formation. In the case of the pyridazine core, an S_N_Ar reaction between 3,5-dichloropyridazine (**23a**) and (*R*)-tetrahydrofuran-3-ol gave the chloride intermediate **24a** which was subsequently converted to ester intermediate **25a** via palladium-catalyzed carbonylation in ethanol (Scheme 2, Route 1). For aryl/heteroaryl alcohols, such as ethyl 5-hydroxynicotinate (**28b**) and ethyl 2,3-difluoro-5-hydroxybenzoate (**28c**), Mitsunobu reactions with (*S*)-tetrahydrofuran-3-ol afforded the alkyl ether intermediates **25b** and **25c** (Scheme 2, Route 2). Finally, chlorides **30e**–**30g** underwent an S_N_Ar reaction with (*R*)-tetrahydrofuran-3-ol to afford ester intermediates **25e**–**25g** (Scheme 2, Route 4). With intermediates **25a**–**25c** and **25e**–**25g** in hand, ester hydrolysis under basic conditions provided carboxylic acid intermediates **26** in quantitative yield. Alternatively, direct nucleophilic aromatic substitution reaction between commercial carboxylic acid **29d** with (*R*)-tetrahydrofuran-3-ol afforded carboxylic acid **26d** in quantitative yield (Scheme 2, Route 3). With the requisite acid intermediates in hand, reactions with phosphorus oxychloride and either 5-fluoropyridin-2-amine or 4-methylthiazol-2-amine in pyridine afforded compounds **27a**–**27g** in low to modest yields. Compounds **19b**–**19g** and **27a**–**27g** were screened against human mGlu_5_ to determine potency with the results highlighted in Table 3.

**Scheme 2:**
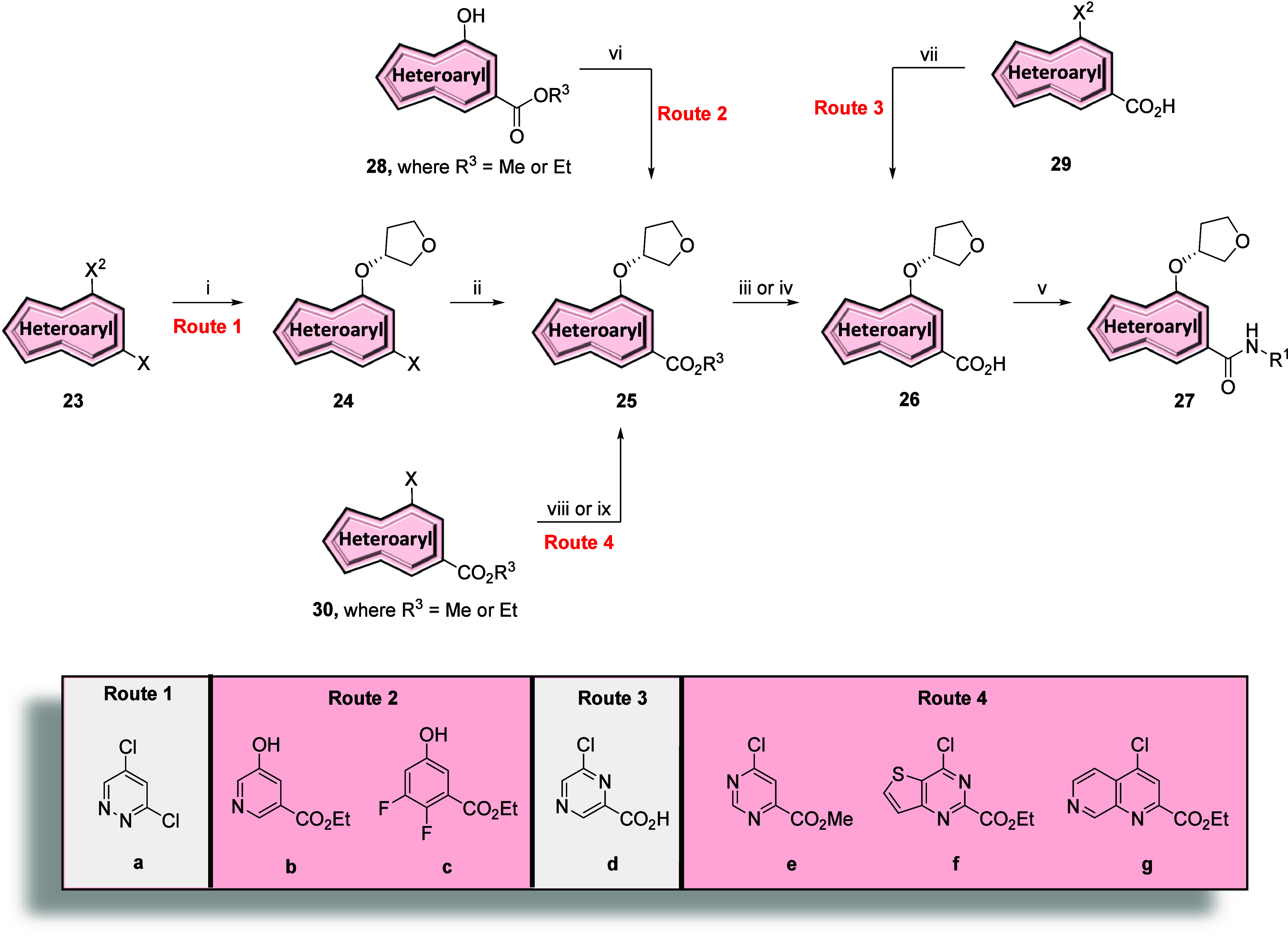
Synthesis of mGlu_5_ NAM Analogues **27a**–**27g** ^a^

a Reagents and conditions: (i) (*R*)-tetrahydrofuran-3-ol, NaH, NMP, 0–23 °C, 1 h, 36%; (ii) Pd­(dppf)­Cl_2_•DCM, NaOAc, EtOH/DMF, CO_(g)_, 70 °C, 3 h, 90%; (iii) 2*M* NaOH_(aq)_ or 2*M* LiOH_(aq)_, THF, 1–2 h, 85%–99%; (iv) 2*M* LiOH_(aq)_, THF, 60 °C, 8 h, 85% (for **30f**); (v) POCl_3_, R^1^NH_2_, pyridine, 23 °C, 30 min, 2%–47%; (vi) (*S*)-tetrahydrofuran-3-ol, DIAD, PPh_3_, THF, 23 °C, 1 h, 62%–74%; (vii) (*R*)-tetrahydrofuran-3-ol, KHMDS, THF, microwave irradiated at 150 °C, 15 min, 99%; (viii) (*R*)-tetrahydrofuran-3-ol, KO^*t*^Bu, DMF, 80 °C, 30 min, 99% (for **30e**); (ix) (*R*)-tetrahydrofuran-3-ol, KHMDS, DMF, 0 °C, 30 min, 58%–72% (for **30f**–**30g**).

**Table 3:**
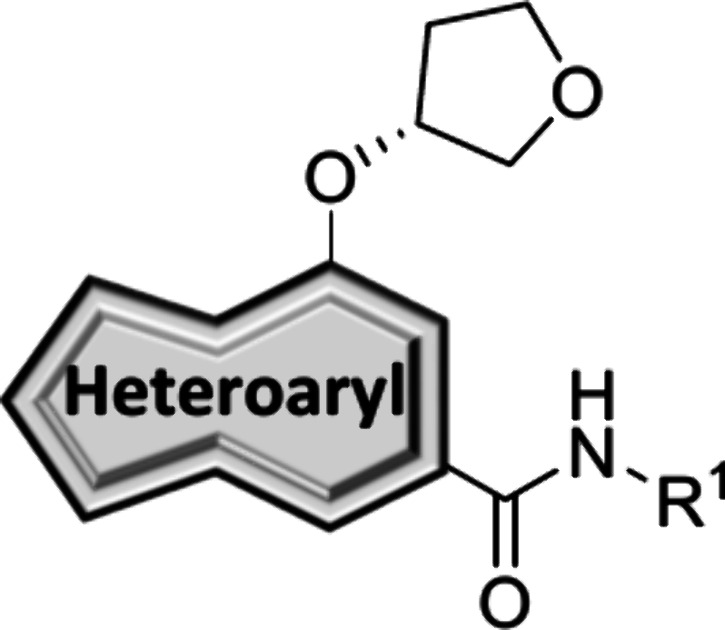

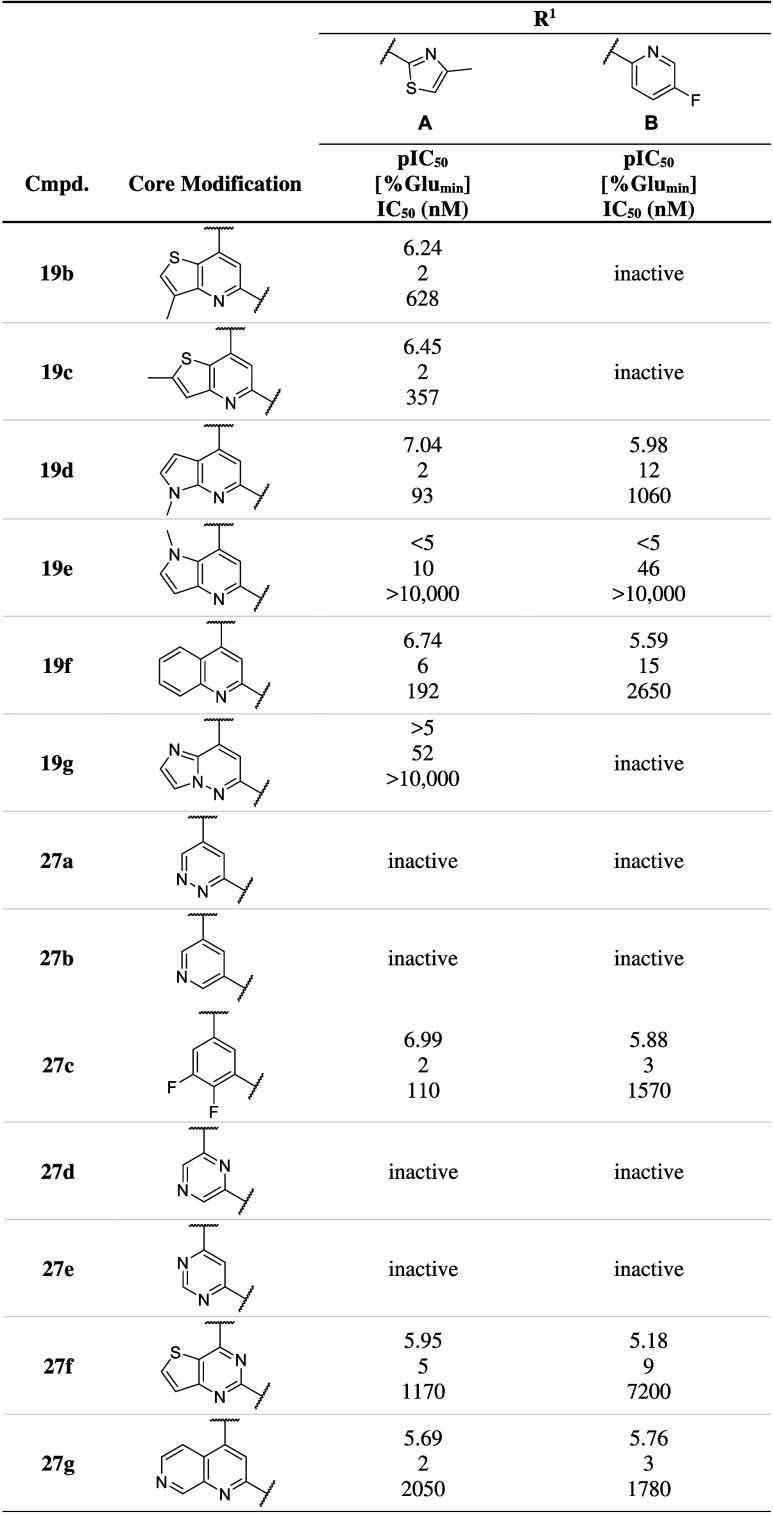
Structures and Activities for Analogues **19b**–**19f** and **27a**–**27g** ^a^

a Calcium mobilization assays in human mGlu_5_-HEK293A cells were performed in the presence of an EC_80_ fixed concentration of glutamate, *n* = 2 independent experiments in triplicate. The % Glu_Min_ is the measure of efficacy of the NAM to reduce an EC_80_ response of glutamate.

We quickly noticed varying the pyridine regiochemistry of the picolinamide core resulted in a complete loss of potency (**27bA-B**). Other 6-membered nitrogen-containing heterocycles including pyridazine (**27aA-B**), pyrazine (**27dA-B**), and pyrimidine (**27eA-B**) were similarly unsuccessful. These results demonstrate the importance of the nitrogen position within the pyridine ring of the picolinamide core. Notably, the 1,2-difluorobenzyl core was a competent picolinamide replacement in the context of the 4-methylthiazole amide (**27cA**: hmGlu_5_ IC_50_ = 110 nM); however, the 5-fluoropyridine amide was 14-fold less potent (**27cB**: hmGlu_5_ IC_50_ = 1.6 μM). Throughout this exercise, this general trend was observed, indicating the importance of the amide tail substitution.

Postulating that the methyl substitution of the pyridine core of compounds **10** and **11** was important for maintaining potency, we returned our attention to bicyclic ring systems to mimic the methyl substitution with alternative 5,6- or 6,6-fused heteroaryl ring systems. While the quinoline core provided a modestly potent analogue in the context of the 4-methylthiazole amide (**19fA**: hmGlu_5_ IC_50_ = 192 nM), the addition of a nitrogen atom to give a 1,7-naphthyridine core was detrimental to potency (**27gA**: hmGlu_5_ IC_50_ = 2.1 μM). Similarly, addition of a second nitrogen to the thieno­[3,2-*b*]­pyridine ring of **19a** to generate the thieno­[3,2-*d*]­pyrimidine also resulted in a loss of potency (**27fA**: hmGlu_5_ IC_50_ = 1.2 μM) as did substitutions on the thieno­[3,2-*b*]­pyridine ring (**19bA**: hmGlu_5_ IC_50_ = 328 nM; **19cA**: hmGlu_5_ IC_50_ = 357 nM). Other core replacements such as 1-methyl-1*H*-pyrrolo­[3,2-*b*]­pyridine (**19e**) and imidazo­[1,2-*b*]­pyridazine (**19g**) proved to be ineffective. Conversely, the 1-methyl-1*H*-pyrrolo­[2,3-*b*]­pyridine core delivered a potent mGlu_5_ NAM (**19dA**: hmGlu_5_ IC_50_ = 93 nM).

To determine which compounds would be advanced into extensive *in vitro* and *in vivo* drug metabolism and pharmacokinetic (DMPK) characterization, we initially evaluated rat predicted hepatic clearance (CL_hep_) and human plasma fraction unbound (*f*_u,plasma_) of our most potent analogues (hmGlu_5_ IC_50_ ≤ 110 nM) as a method to quickly triage compounds (results highlighted in Table 4). Many of the analogues displayed high rat predicted CL_hep_ based on *in vitro* microsomal data (>47 mL/min/kg); however, analogues **19aA**, **19aL**, and **27cA** were predicted to have moderate rat CL_hep_ (44–47 mL/min/kg), compared to the predecessor compound **VU0604545** (**11**) (CL_hep_ = 51 mL/min/kg). Of these three compounds, **19aA** (*f*_u,plasma_ = 0.020) and **27cA** (*f*_u,plasma_ = 0.069) were determined to have the most attractive human plasma fraction unbound. Based on these data, **19aA** (**VU6031545**) and **27cA** (**VU6024945**) were selected to advance into a battery of *in vitro* and *in vivo* DMPK assays and our standard rat plasma:brain level (PBL) cassette studies (Table 5).

**Table 4:** *In Vitro* Predicted Rat Hepatic Clearance (CL_hep_) and Human Plasma Fraction Unbound (*f*_u,plasma_) of the Most Potent mGlu_5_ NAMs

compound	IC_50_ (nM)	rat CL_hep_ (mL/min/kg)	human *f*_u,plasma_
**19aA**	15	47.0	0.020
**19aB**	61	55.1	0.008
**19aD**	22	57.5	0.005
**19aL**	55	41.8	0.016
**19dA**	93	52.3	0.006
**22i**	99	48.2	0.005
**22j**	107	68.4	0.001
**27cA**	110	43.7	0.069

**Table 5:** *In Vitro* and *In Vivo* DMPK Data for Analogues **19aA** and **27cA**

property	**19aA**, **VU6031545**	**27cA**, **VU6024945**
MW	361.43	340.34
*x* log *P*	3.23	3.23
TPSA	73.3	60.4
***In Vitro* PK Parameters**		
CL_int_ (mL/min/kg), rat	143	116
CL_hep_ (mL/min/kg), rat	47.0	43.7
CL_int_ (mL/min/kg), human	140	241
CL_hep_ (mL/min/kg), human	18.3	19.3
rat *f*_*u*,plasma_ ^a^	0.027	0.040
human *f*_*u*,plasma_ ^a^	0.020	0.069
Rat *f*_*u*,brain_ ^a^	0.009	0.051
***In Vivo* PK Parameters** ^b^		
CL_p_ (mL/min/kg)	49.3	31.3
Elim. *t*_1/2_ (h)	3.42	1.78
MRT (h)	2.45	1.44
*V*_ss_ (L/kg)	6.67	2.89
%F^c^	>100	16.6
**Brain Distribution (0.25 h) (SD Rat; 0.2** mg/kg IV)		
*K*_p brain:plasma_ ^c^	3.73	0.67
*K*_p,uu brain:plasma_ ^d^	1.29	0.84
**CYP**_**450**_ **IC**_**50**_ **(μM)**		
**1A2**	1.2	1.3
**2C9**	>30	>30
**2D6**	>30	>30
**3A4**	>30	>30

a *f*_*u*_ = fraction unbound; equilibrium dialysis assay; brain = rat brain homogenates.

b Male Sprague–Dawley rats (*n* = 2); IV PK: 1 mg/kg, vehicle = 10% ethanol, 40% PEG400, 50% saline; PO PK: 10 mg/kg, vehicle = 10% Tween80 in water.

c *K*_p_ = total brain-to-plasma partition ratio.

d *K*_p,uu_ = unbound brain-to-plasma partition ratio [(brain *f*_*u*_ × total brain)/(plasma *f*_*u*_ × total plasma)].

Regarding physicochemical properties, both compounds possessed molecular weights less than 365 Da with attractive *x* Log *P* values (<4) for CNS penetration. Although both compounds were predicted to have moderate clearance in rats, both were predicted to have high clearance in human (CL_hep_ > 15 mL/min/kg). While both compounds exhibited moderate plasma free faction in rat, **VU6024945** demonstrated a more desirable fraction unbound in rat brain homogenates (*f*_u,brain_ = 0.051) versus **VU6031545** (*f*_u,brain_ = 0.009). Although **VU6024945** displayed sufficient CNS distribution of unbound drug (rat brain:plasma *K*_p_ = 0.67; *K*_p,uu_ = 0.84), **VU6031545** proved to have higher CNS penetration (rat brain:plasma *K*_p_ = 3.73; *K*_p,uu_ = 1.29). When cytochrome P450 (CYP450) inhibition was evaluated, both analogues displayed a CYP1A2 IC_50_ ≤ 1.3 μM with no appreciable inhibition observed for the other isoforms tested (CYP 2C9, 2D6, 3A4 IC_50_ > 30 μM).

Due to the previous IVIVC disconnect observed for the **VU0604545** (**11**) series, we accessed both compounds in *in-vivo* IV/PO PK experiments. Gratifyingly, the predicted rat clearance of both **VU6031545** (CL_hep_ = 47.0 mL/min/kg) and **VU6024945** (CL_hep_ = 43.7 mL/min/kg) were in good agreement with the *in-vivo* clearances (CL_p_ = 49.3 mL/min/kg and CL_p_ = 31.3 mL/min/kg, respectively). **VU6031545** displayed a high volume of distribution (*V*_ss_ = 6.67 L/kg) with an elimination half-life of 3.42 h and an oral bioavailability >100%. **VU6024945** demonstrated a moderate volume of distribution (*V*_ss_ = 2.89 L/kg) with an elimination half-life of 1.78 h and a lower oral bioavailability (%F = 17). Both compounds represent an improvement over the previous series (**11**), which showed an IVIVC disconnect, a short elimination half-life (*t*_1/2_ = 46 min), and poor oral bioavailability (%F = 5.5). Moreover, when compared to the structurally similar predecessor analogue **VU0409106** (CYP1A2 IC_50_ < 100 nM, %*F* < 5%), an analogue of **11** bearing a fluorophenyl core and methyl thiazole amide tail, incorporation of a sp^3^-hybridized headgroup to provide **VU6024945** afforded improving drug-like properties (CYP1A2 IC_50_ = 1.3 μM, %*F* = 17%).^22^

In conclusion, novel mGlu_5_ NAMs were identified utilizing a scaffold hopping approach. Our new generation of mGlu_5_ NAMs lack the classical aryl/heterobiaryl acetylene chemotype which has been linked to poor PK and hepatotoxicity. Utilizing thieno­[3,2-*b*]­pyridine-5-carboxamide as a core replacement for 6-methylpicolinomide of **VU0604545** (**11**), we were able to identify several potent mGlu_5_ NAMs (hmGlu_5_ IC_50_ < 80 nM). A follow-up exercise explored alternate cores and resulted in the discovery of additional highly potent mGlu_5_ NAMs (hmGlu_5_ IC_50_ ≤ 110 nM). Although many of these potent analogues displayed high predicted rat CL_hep_ and/or high plasma protein binding in human, two compounds (**19aA** and **27cA**) displayed moderate predicted clearance and moderate plasma fraction unbound in human. Both compounds were advanced into further DMPK profiling. **VU6031545** (**19aA**) proved to be highly CNS penetrant (*K*_p_ = 3.73) with a high distribution of unbound drug (*K*_p,uu_ = 1.29) which further improved upon predecessor compound **VU0604545** (**11**) (rat brain:plasma *K*_p,uu_ = 0.79). Additionally, both **VU6031545** (**19aA**) and **VU6024945** (**27cA**) showed improvement in elimination half-life and oral bioavailability when compared to predecessor **VU0604545** (**11**). Unlike the previous series, these compounds showed good IVIVC. Unfortunately, both compounds were predicted to have high clearance in human (CL_hep_ > 15 mL/min/kg) and inhibited CYP1A2 (IC_50_ ≤ 1.3 μM) and further progression was halted. While our current endeavor did not generate mGlu_5_ NAMs with suitable DMPK profiles to warrant further development, it did provide invaluable SAR insights for future scaffold designs. Further refinements are underway and will be reported in due course.

## Supplementary Material



## Notes

General methods for the synthesis and characterization of key compounds and experimental details for calcium mobilization assays, *in vitro* and *in vivo* DMPK protocols (PDF)
