# Supplementary material for: Discovery of Thieno[3,2‑b]pyridine-5-carboxamide and 2,3-Difluorobenzamide Negative Allosteric Modulators of Metabotropic Glutamate Receptor Subtype 5
Source: ACS Med Chem Lett. 2025 Apr 22;16(5):865–74. doi: 10.1021/acsmedchemlett.5c00119 (PMC12067350; doi:10.1021/acsmedchemlett.5c00119)
Supplement: Supplementary file 1 [file ml5c00119_si_001.pdf]

Supplemental Information for

Discovery of Thieno[3,2-*b*]pyridine-5-carboxamide and 2,3-Difluorobenzamide Negative Allosteric Modulators of Metabotropic Glutamate Receptor Subtype 5

Katherine E. Crocker[a,b*†], Scott H. Henderson[a,b†], Rory A. Capstick[a,b], David L. Whomble[a,b], Aaron M. Bender[a,b], Andrew S. Felts[a,b], Changho Han[a,b], Julie L. Engers[a,b], Natasha B. Billard[a,b], Mallory A. Maurer[a,b], Hyekyung P. Cho[a,b], Alice L. Rodriguez[a,b], Colleen M. Niswender[a,b,d,e,f], Jordan O'Neill[a,b], Katherine J. Watson[a,b], Sichen Chang[a,b], Anna L. Blobaum[a,b], Olivier Boutaud[a,b], Weimin Peng[b], Jerri M. Rook[b], P. Jeffrey Conn[a,b,f], Craig W. Lindsley[a,b,c,d], Kayla J. Temple[a,b*]

[a]Warren Center for Neuroscience Drug Discovery, Vanderbilt University, Nashville, TN 37232, USA
[b]Department of Pharmacology, Vanderbilt University School of Medicine, Nashville, TN 37232, USA
[c]Department of Chemistry, Vanderbilt University, Nashville, TN 37232, USA
[d]Department of Biochemistry, Vanderbilt University, Nashville, TN 37232, USA
[e]Vanderbilt Kennedy Center, Vanderbilt University School of Medicine, Nashville, TN 37232, USA
[f]Vanderbilt Brain Institute, Vanderbilt University School of Medicine, Nashville, TN 37232, USA
†Authors contributed equally to this work
*Corresponding authors' email:
kayla.temple@vanderbilt.edu
katherine.crocker@vanderbilt.edu

Table of Contents

Experimental Synthetic Procedures and Spectroscopic Data S2
General Synthetic Methods........ S2
General Instrumentation Methods. S2
General Procedure for the Preparation of Analog 19aA........ S4
General Procedure for the Preparation of Analog 27cA........ S5
NMR Spectra S8
Molecular Pharmacology Methods........ S12
Calcium Mobilization Assays........ S12
DMPK Methods........ S13
IV plasma-brain level determination (PBL) and pharmacokinetics (PK). S13
Binding in plasma from rat and human. S15
Binding in brain homogenate from rat........ S15
Intrinsic Clearance in Rat and Human Liver Microsomes S16
Cytochrome P450 inhibition using Liver Microsomes........ S17
LC-MS/MS Analysis S17

Experimental Synthetic Procedures and Spectroscopic Data

General Synthetic Methods.

All reactions were carried out employing standard chemical techniques. Solvents used for extraction, washing, and chromatography were HPLC grade. All reagents were purchased from commercial sources and were used without further purification.

Automated flash column chromatography was performed on a Biotage Isolera 1 or a Teledyne ISCO CombiFlash system. RP-HPLC was performed on a Gilson preparative reversed-phase HPLC system comprised of a 333 aqueous pump with solvent-selection valve, 334 organic pump, GX-271 or GX-281 liquid hander, two column switching valves, and a 155 UV detector. Absorbance was typically monitored at 215 or 220 nm. Column: Phenomenex Axia-packed Gemini C18, 5 μm. Mobile phase: CH_3CN in H_2O (0.1% TFA) or CH_3CN in H_2O (0.05% *v/v* NH_4OH) under the specified gradient, then hold 95% CH_3CN in 5% aqueous phase, 50 mL/min, 23° C. All compounds were found to be >95% pure by LCMS analysis.

Safety statement*: no unexpected or unusually high safety hazards were encountered.*

General Instrumentation Methods.

All NMR spectra were recorded on a 400 MHz AMX Bruker NMR spectrometer. ^{1}H and ^{13}C chemical shifts are reported in δ values in ppm downfield with the deuterated solvent as the internal standard. Data are reported as follows: chemical shift, multiplicity (s = singlet, d = doublet, t = triplet, q = quartet, b = broad, m = multiplet), integration, coupling constant (Hz).

Low resolution mass spectra (LRMS) were obtained on an Agilent 6120/6150 or Waters QDa (Performance) SQ MS with ESI source. *Method A (Agilent 6120/6150):* MS parameters were as follows: fragmentor: 70, capillary voltage: 3000 V, nebulizer pressure: 30 psig, drying gas flow: 13 L/min, drying gas temperature: 350 ºC. Samples were introduced via an Agilent 1290 UHPLC comprised of a G4220A binary pump, G4226A ALS, G1316C TCC, and G4212A DAD with ULD flow cell. UV absorption was generally observed at 215 nm and 254 nm with a 4 nm bandwidth. Column: Waters Acquity BEH C18, 1.0 x 50 mm, 1.7 um. Gradient conditions: 5% to 95% CH_3CN in H_2O (0.1% TFA) over 1.4 min, hold at 95% CH_3CN for 0.1 min, 0.5 mL/min, 55 ºC. *Method B (Agilent 6120/6150):* MS parameters were as follows: fragmentor: 100, capillary voltage: 3000 V, nebulizer pressure: 40 psig, drying gas flow: 11 L/min, drying

gas temperature: 350 ºC. Samples were introduced via an Agilent 1200 HPLC comprised of a degasser, G1312A binary pump, G1367B HP-ALS, G1316A TCC, G1315D DAD, and a Varian 380 ELSD (if applicable). UV absorption was generally observed at 215 nm and 254 nm with a 4 nm bandwidth. Column: Thermo Accucore C18, 2.1 x 30 mm, 2.6 um. Gradient conditions: 7% to 95% CH_3CN in H_2O (0.1% TFA) over 1.6 min, hold at 95% CH_3CN for 0.35 min, 1.5 mL/min, 45 ºC. *Method C (Waters QDa (Performance) SQ MS)*: MS parameters were as follows: cone voltage: 15 V, capillary voltage: 0.8 kV, probe temperature: 600° C. Samples were introduced via an Acquity I-Class PLUS UPLC comprised of a BSM, FL-SM, CH-A, and PDA. UV absorption was generally observed at 215 nm and 254 nm; 4 nm bandwidth. Column: Phenomenex EVO C18, 1.0 x 50 mm, 1.7 um. Column temperature: 55° C. Flow rate: 0.4 mL/min. Default gradient: 5% to 95% CH_3CN (0.05% TFA) in H_2O (0.05% TFA) over 1.4 min (curve 6), hold at 95% CH_3CN for 0.1 min. "Polar" (2% to 70% CH_3CN (0.05% TFA) in H_2O (0.05% TFA) over 0.8 min (curve 6), transition to 95% CH_3CN over 0.1 min (curve 6), hold at 95% CH_3CN for 0.6 min.) and "Non-Polar" (40% to 95% CH_3CN (0.05% TFA) in H_2O (0.05% TFA) over 1.4 min (curve 6), hold at 95% CH_3CN for 0.1 min.) gradients were also available. *Method D (Waters QDa (Performance) SQ MS):* MS parameters were as follows: cone voltage: 15 V, capillary voltage: 0.8 kV, probe temperature: 600° C. Samples were introduced via an Acquity I-Class PLUS UPLC comprised of a BSM, FL-SM, CH-A, and PDA. UV absorption was generally observed at 215 nm and 254 nm with a 4 nm bandwidth. Column: Phenomenex EVO C18, 1.0 x 50 mm, 1.7 um. Column temperature: 55° C. Flow rate: 0.4 mL/min. Default gradient: 5% to 95% CH_3CN in H_2O (5 mM NH_4HCO_3) over 1.4 min (curve 6), hold at 95% CH_3CN for 0.1 min. "Polar" (2% to 70% CH_3CN in H_2O (5 mM NH_4HCO_3) over 0.8 min (curve 6), transition to 95% CH_3CN over 0.1 min (curve 6), hold at 95% CH_3CN for 0.6 min.) and "Non-Polar" (40% to 95% CH_3CN in H_2O (5 mM NH_4HCO_3) over 1.4 min (curve 6), hold at 95% CH_3CN for 0.1 min.) gradients were also available.

High resolution mass spectra (HRMS) were obtained on an Agilent 6540 UHD Q-TOF with ESI source. MS parameters were as follows: fragmentor: 150, capillary voltage: 3500 V, nebulizer pressure: 60 psig, drying gas flow: 13 L/min, drying gas temperature: 275 ºC. Samples were introduced via an Agilent 1200 UHPLC comprised of a G4220A binary pump, G4226A 3 ALS, G1316C TCC, and G4212A DAD with ULD flow cell. UV absorption was observed at 215 nm and 254 nm with a 4 nm bandwidth. Column: Agilent Zorbax Extend C18, 1.8 µm, 2.1 x 50 mm. Gradient conditions: 5% to 95% CH_3CN in H_2O (0.1% formic acid) over 1 min, hold at 95% CH_3CN for 0.1 min, 0.5 mL/min, 40 ºC.

General Procedure for the Preparation of Analog 19aA.

Synthesis of Intermediate 17a (Scheme 1):

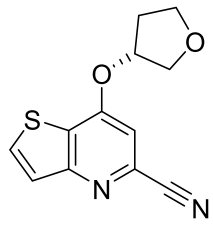

(*R*)-Tetrahydrofuran-3-ol (0.27 mL, 3.4 mmol) was dissolved in DMF (1.5 mL) and KHMDS (1.41 mL, 6.5 mmol) was added. The reaction was cooled to 0 ℃ and 7-chlorothieno[3,2-*b*]pyridine-5-carbonitrile (600 mg, 3.1 mmol) was added as a solution in DMF (1 mL) and the reaction was stirred for 30 minutes. Saturated aqueous NH_4Cl was added and the reaction extracted with EtOAc (3x). The combined organic layers were washed with brine, dried (Na_2SO_4), filtered and concentrated *in vacuo*. Purification by flash chromatography on silica gel using 0-40% EtOAc/hexanes afforded 700 mg of the title compound (90% yield). ^{1}H NMR (400 MHz, $CDCl_3$) δ 7.84 (d, *J* = 5.5 Hz, 1H), 7.55 (d, *J* = 5.5 Hz, 1H), 6.99 (s, 1H), 5.24 – 5.17 (m, 1H), 4.12 (d, *J* = 3.2 Hz, 2H), 4.05 (td, *J* = 8.4, 6.8 Hz, 1H), 3.97 (td, *J* = 8.4, 4.4 Hz, 1H), 2.39 (dtd, *J* = 14.3, 8.3, 6.2 Hz, 1H), 2.25 (dddd, *J* = 13.5, 6.5, 4.4, 1.6 Hz, 1H). LRMS: $C_{12}H_{10}N_2O_2S$ $[M+H]^+$ calc. mass 247.1, found 247.4.

Synthesis of Intermediate 18a (Scheme 1):

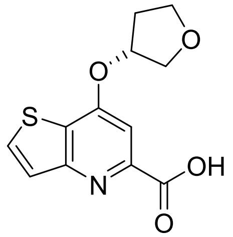

To a solution of (*R*)-7-((tetrahydrofuran-3-yl)oxy)thieno[3,2-*b*]pyridine-5-carbonitrile (700 mg, 2.84 mmol) in 1,4-dioxane (2.8 mL) was added 2*N* NaOH (7.1 mL, 14.2 mmol). The mixture was heated at 100 °C in a sealed vial for 18 hours. After cooling, the pH of the reaction was adjusted to pH 4-5 using 1*N* HCl and the mixture was concentrated *in vacuo*. The crude residue was dissolved in 10% MeOH/CH_2Cl_2, filtered to remove the insoluble salts, and the filtrate concentrated *in vacuo* to afford 754 mg (100% yield) of the title compound which was used without further purification. ^{1}H NMR (400 MHz, CD_3OD) δ 8.58 (d, *J* = 5.6 Hz, 1H), 7.97 (s, 1H), 7.81 (d, *J* = 5.6 Hz, 1H), 5.83 – 5.76 (m, 1H), 4.19 (dt, *J* = 11.0, 1.2 Hz, 1H), 4.15 – 4.02 (m, 2H), 3.96 (td, *J* = 8.5, 4.6 Hz, 1H), 2.55 (dtd, *J* = 14.2, 8.2, 6.0 Hz, 1H), 2.33 (dddt, *J* =

14.2, 7.2, 4.7, 1.3 Hz, 1H), (OH proton not observed). LRMS: $C_{12}H_{11}NO_4S$ $[M+H]^+$ calc. mass 266.0, found 266.1.

Preparation of Compound ***19aA (VU6031545)*** *(Scheme 1):*

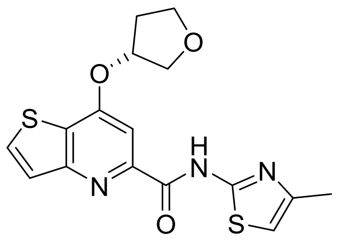

(*R*)-7-((Tetrahydrofuran-3-yl)oxy)thieno[3,2-*b*]pyridine-5-carboxylic acid (130 mg, 0.49 mmol) and 4-methylthiazol-2-amine (84 mg, 0.74 mmol) were dissolved in pyridine (5 mL). Phosphorus(V) oxychloride (69 μL, 0.74 mmol) was added dropwise and the reaction was allowed to stir for 30 minutes. Water (0.1 mL) was added and the reaction concentrated *in vacuo*. Crude product was dissolved in DMSO (3 mL) and purified using RP-HPLC (25 - 80% ACN/0.1% aqueous TFA). Fractions containing product were basified with a saturated aqueous $NaHCO_3$ solution and extracted with EtOAc (3x). Solvents were concentrated to give 27 mg (15% yield) of the title compound. ^1H NMR (400 MHz, $CDCl_3$) δ 11.29 (s, 1H), 7.81 (d, J = 5.5 Hz, 1H), 7.66 (s, 1H), 7.51 (d, J = 5.5 Hz, 1H), 6.62 (d, J = 1.2 Hz, 1H), 5.36-5.25 (m, 1H), 4.14 (d, J = 3.2 Hz, 2H), 4.05 (dt, J = 8.5, 6.8 Hz, 1H), 3.97 (td, J = 8.4, 4.3 Hz, 1H), 2.46 – 2.34 (m, 4H), 2.34 – 2.24 (m, 1H). ^{13}C NMR (101 MHz, $CDCl_3$) δ 162.66, 160.35, 157.71, 157.05, 147.89, 146.97, 132.36, 126.43, 125.32, 108.50, 99.77, 79.39, 77.36, 73.04, 67.40, 33.22. HRMS: $C_{16}H_{15}N_3O_3S_2$ $[M+H]^+$ calc. mass 362.0628, found 362.0630.

General Procedure for the Preparation of Analog 27cA.

Synthesis of Intermediate 25c (Scheme 2):

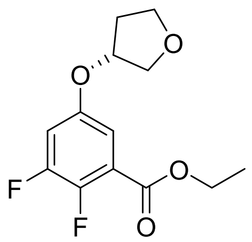

Ethyl 2,3-difluoro-5-hydroxybenzoate (42 mg, 0.21 mmol), (*S*)-tetrahydrofuran-3-ol (212 mg, 0.25 mmol) and PPh_3 (65 mg, 0.25 mmol) were dissolved in THF (2 mL) and DIAD (49 μL, 0.25 mmol) was added

dropwise. The reaction was stirred for 1 hour and then concentrated *in vacuo*. Purification via normal-phase column chromatography on silica gel (3-40% EtOAc/hexanes) gave 34 mg (62% yield) of title compound. ^{1}H NMR (400 MHz, $CDCl_3$) δ 7.16 – 7.10 (m, 1H), 6.91 – 6.86 (m, 1H), 4.92 – 4.86 (m, 1H), 4.40 (q, J = 7.1 Hz, 2H), 4.01 – 3.88 (m, 4H), 2.28 -2.19 (m, 1H), 2.15 – 2.07 (m, 1H), 1.40 (t, J = 7.1 Hz, 3H); LRMS: $C_{13}H_{14}F_2O_4$ $[M+H]^+$ calc. mass 273.1, found 273.2.

Synthesis of Intermediate 26c (Scheme 2):

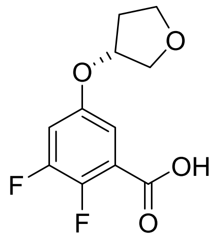

To a solution of ethyl (*R*)-2,3-difluoro-5-((tetrahydrofuran-3-yl)oxy)benzoate (33 mg, 0.12 mmol) in THF (1 mL) was added LiOH (9 mg, 0.37 mmol) in water (1 mL). The mixture was stirred for 1 hour and then the pH of the reaction was adjusted to pH 2-3 using 2*N* HCl and the mixture extracted with DCM (3x). The combined organic washes were dried ($MgSO_4$), filtered and concentrated *in vacuo* afford 25 mg (85% yield) of the title compound which was used without further purification. ^{1}H NMR (400 MHz, DMSO-*d*$_6$) δ 7.38 – 7.33 (m, 1H), 7.11 – 7.09 (m, 1H), 5.10 – 5.05 (m, 1H), 3.87 – 3.72 (m, 4H), 2.26 – 2.17 (m, 1H), 1.98 – 1.91 (m, 1H) (OH proton not observed).

Preparation of Compound ***27cA (VU6024945)*** *(Scheme 2):*

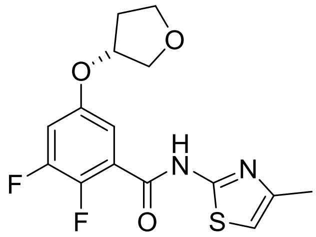

(*R*)-2,3-Difluoro-5-((tetrahydrofuran-3-yl)oxy)benzoic acid (635 mg, 2.6 mmol) and 4-methylthiazol-2-amine (446 mg, 3.9 mmol) were dissolved in pyridine (13 mL) then cooled to 0 °C. Phosphorus(V) oxychloride (364 µL, 3.9 mmol) was added dropwise and the reaction was allowed to warm to room temperature over 30 minutes. Water (1 mL) was added and the reaction concentrated *in vacuo*. The resulting residue was partitioned between water and DCM and the layers separated. The aqueous layer was further extracted with DCM (2x) and the combined organic extracts were dried ($MgSO_4$), filtered and concentrated

in vacuo. Crude product was dissolved in DMSO (5 mL) and purified using RP-HPLC (25 - 75% ACN/0.1% aqueous TFA). Fractions containing product were basified with a saturated aqueous $NaHCO_3$ solution and extracted with DCM (3x). Solvents were concentrated to give 186.2 mg (21% yield) of the title compound. ^{1}H NMR (400 MHz, $CDCl_3$) δ 9.84 (s, 1H), 7.37 (dd, J = 5.0, 1.3 Hz, 1H), 6.95 (ddd, J = 10.9, 6.7, 3.2 Hz, 1H), 6.61 (q, J = 1.0 Hz, 1H), 4.94 (td, J = 4.3, 2.1 Hz, 1H), 4.05 – 3.86 (m, 4H), 2.37 (d, J = 1.0 Hz, 3H), 2.26 (dtd, J = 13.6, 8.3, 6.1 Hz, 1H), 2.18 – 2.07 (m, 1H). ^{13}C NMR (101 MHz, $CDCl_3$) δ 160.32 (t, J_{CF} = 3.3 Hz), 157.84, 154.58 (dd, J_{CF} = 9.6, 2.3 Hz), 151.81 (dd, J_{CF} = 250.8, 15.8 Hz), 148.04, 144.91 (dd, J_{CF} = 245.7, 14.9 Hz), 121.65 (d, J_{CF} = 8.8 Hz), 111.62 (d, J_{CF} = 20.1 Hz), 111.10 (d, J_{CF} = 2.9 Hz), 109.93, 79.42, 73.67, 68.02, 33.67, 17.70. HRMS: $C_{15}H_{14}F_2N_2O_3S$ $[M+H]^+$ calc. mass 341.0766, found 341.0766.

NMR Spectra

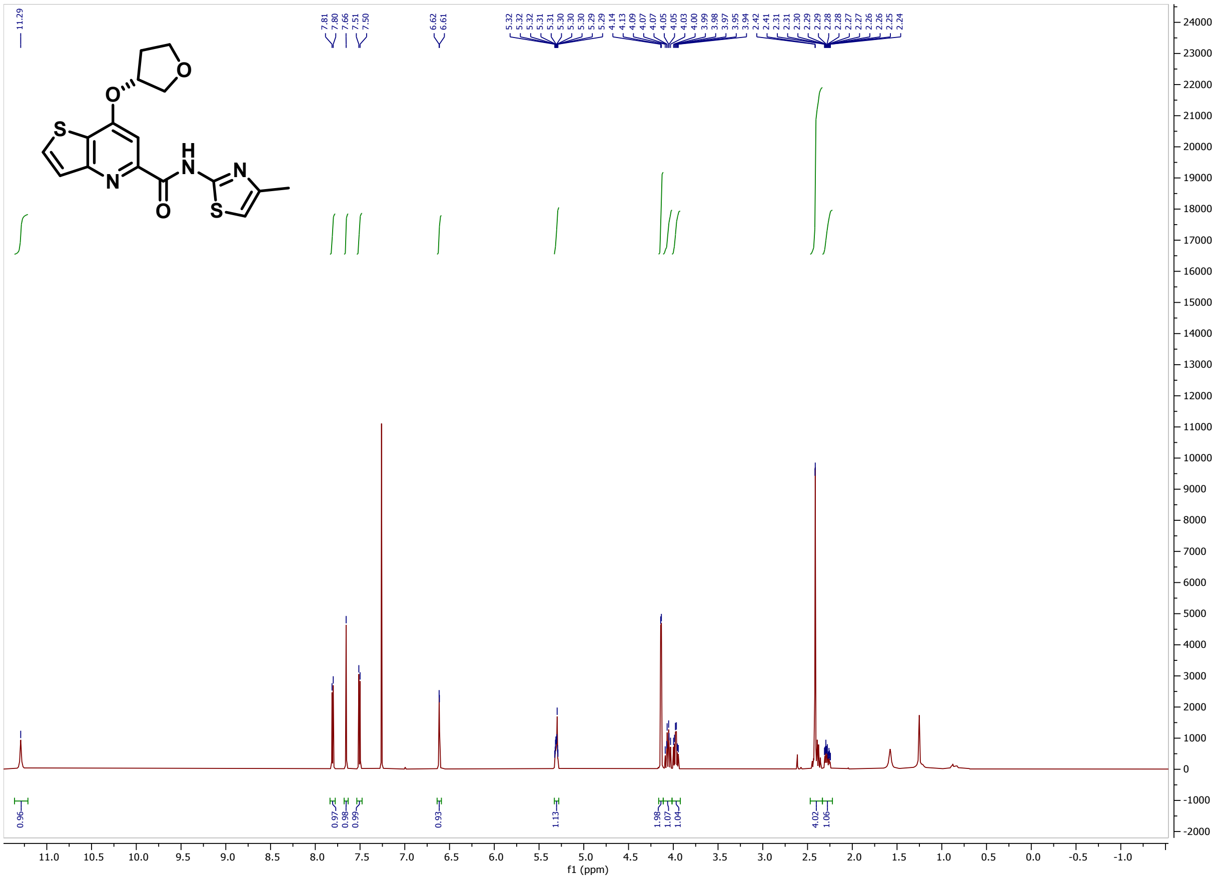

^{1}H NMR Spectra (400 MHz, $CDCl_3$) of **VU6031545** (**19aA**).

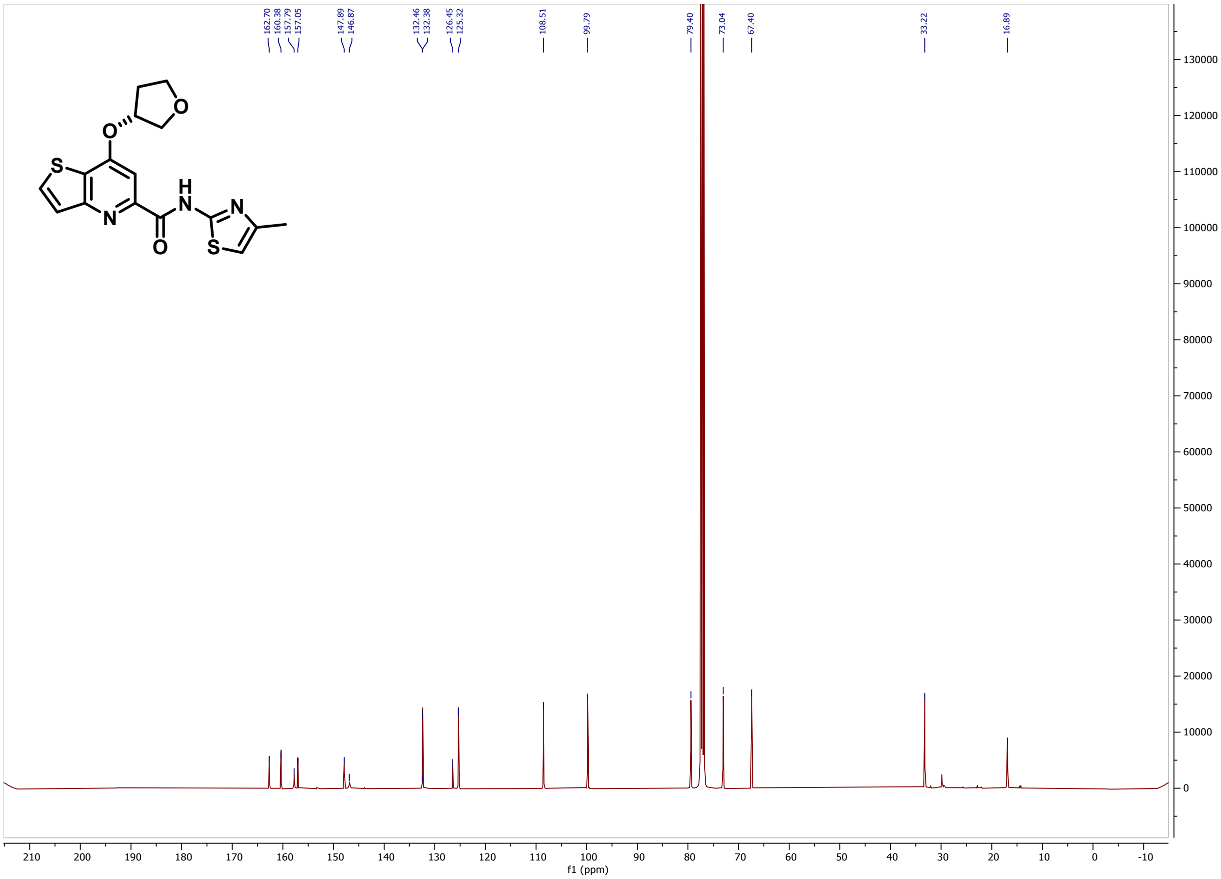

^{13}C NMR Spectra (101 MHz, $CDCl_3$) of **VU6031545** (**19aA**).

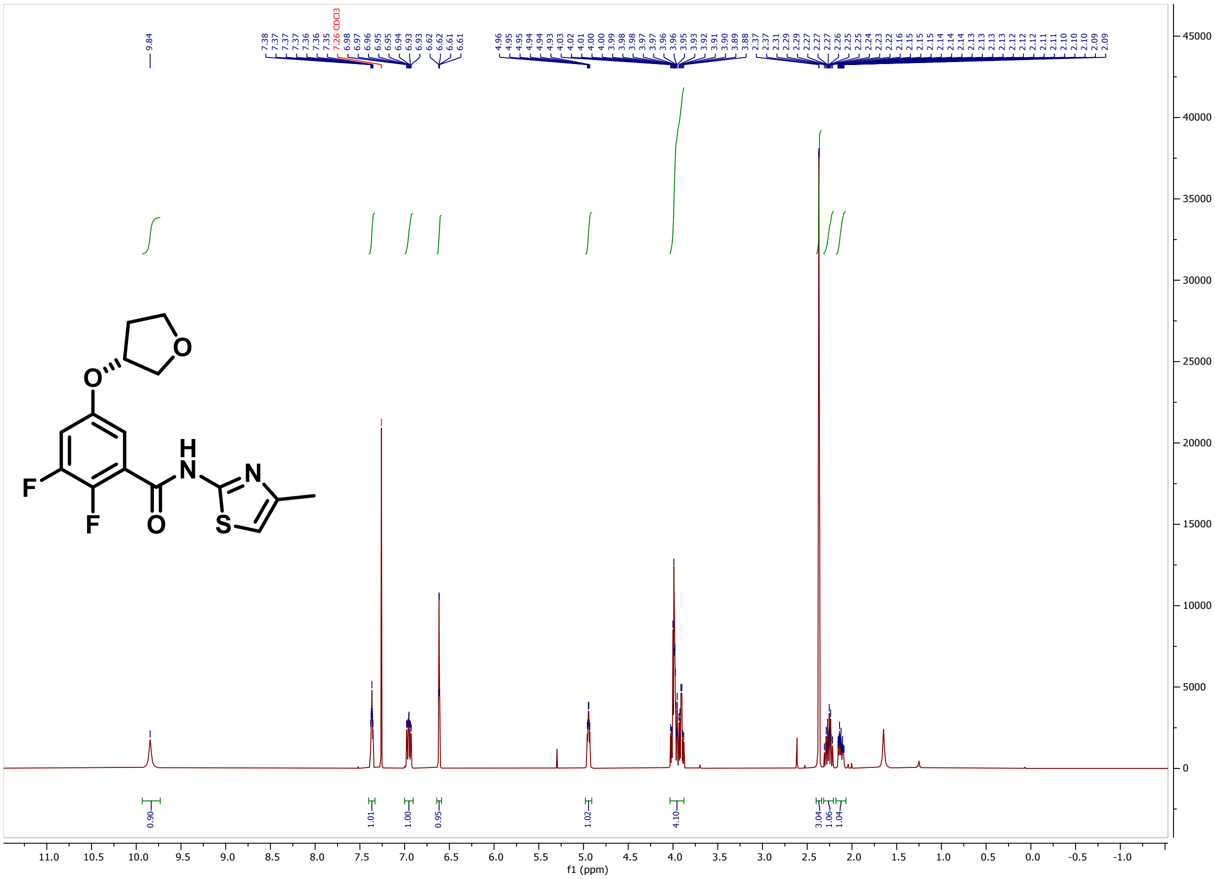

^{1}H NMR Spectra (400 MHz, $CDCl_3$) of **VU6024945** (**27cA**).

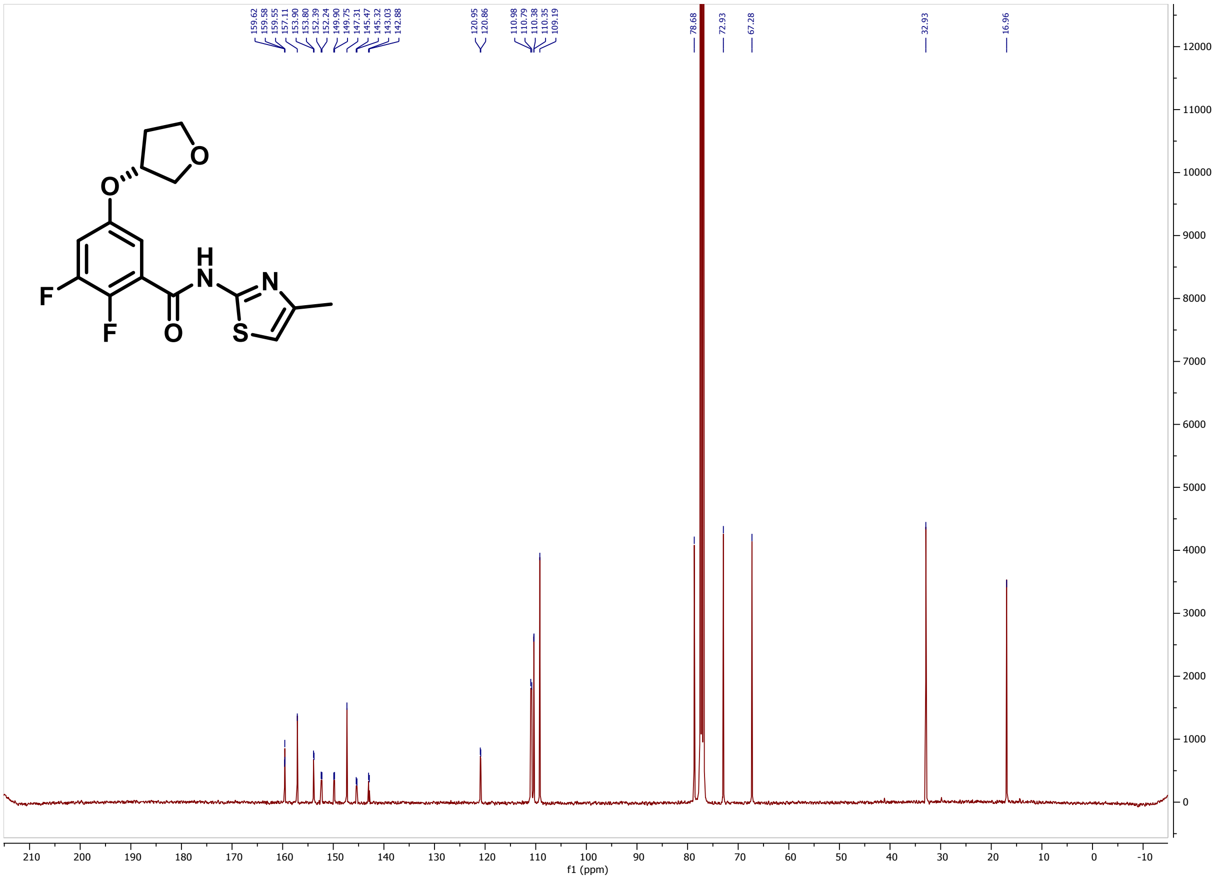

^{13}C NMR Spectra (101 MHz, $CDCl_3$) of **VU6024945** (**27cA**).

Molecular Pharmacology Methods

Calcium Mobilization Assays.

To measure the functional activity of negative allosteric modulator (NAM) compounds in a cellular assay, human metabotropic Glutamate receptor subtype 5 ($mGlu_5$) was stably expressed in the human embryonic kidney (HEK293A) cells to evoke a decrease in intracellular calcium to an EC_{80} concentration of glutamate (Glu) agonist. The stable $mGlu_5$-HEK293A cells were cultured in DMEM medium containing 10% fetal bovine serum, 20 mM HEPES, 2 mM glutamine, 1 mM sodium pyruvate, non-essential amino acid mixture, 0.5 mg/ml G418, and antibiotics/antimycotic. All reagents used were from Life Technologies (Carlsbad, CA) unless otherwise noted.

Briefly, the day before the assay, stable $mGlu_5$-HEK293A cells (20,000 cells/20 μL/well) were plated in in black-walled, clear-bottomed, amine-treated 384 well plates (Corning) in the assay medium (DMEM containing 10% dialyzed fetal bovine serum, 20 mM HEPES, 1 mM sodium pyruvate, and antibiotics/antimycotic). Cells were incubated overnight at 37 °C in the presence of 5% CO2. The next day, calcium assay buffer (Hank's balanced salt solution (HBSS), 20 mM HEPES, 2.5 mM Probenecid, 4.16 mM sodium bicarbonate (Sigma-Aldrich, St. Louis, MO)) was prepared to dilute compounds, agonists, and Fluo-4-acetomethoxyester (Fluo-4-AM, Ion Biosciences), fluorescent calcium indicator dye. Compounds were serially diluted 1:3 into 10 point concentration response curves in DMSO using the Bravo Liquid Handler (Agilent, Santa Clara, CA), transferred to a 384 well daughter plates using an Echo acoustic liquid handler (Beckman Coulter, Indianapolis, Indiana), and diluted in assay Buffer to a 2X final concentration. The agonist plates were prepared using glutamate (Tocris) concentrations for the EC_{20}, EC_{80} and EC_{MAX} responses by diluting in assay buffer to a 5X final concentration. The 2X dye solution (2.3 μM) was prepared by mixing a 2.3 mM Fluo-4-AM stock in DMSO with 10% (w/v) pluronic acid F-127 in a 1:1 ratio in assay buffer. Using a microplate washer (BioTek, Winooski, VT), cells were washed with assay buffer for 3 times to remove medium. After the final wash, 20 μL of assay buffer remained in the cell plates. Immediately, 20 μL of the 2X dye solution (final 1.15 μM) was added to each well of the cell plate using a Multidrop Combi dispenser (Thermo Fisher, Waltham, MA). After cells were incubated with the dye solutions for 45 min at 37 °C in the presence of 5% CO_2, the dye solutions were removed and replaced with assay buffer using a microplate washer, leaving 20 μL of assay buffer in the cell plate. The compound, agonist, and cell plates were placed inside the Functional Drug Screening System (FDSS 7000 or uCell, Hamamatsu, Japan) to measure the calcium flux. The triple add protocol was used to measure Ca kinetics; Compound, Glu for EC_{20}, Glu for $EC_{80/Max}$ adds in an order. Briefly, after establishment of a fluorescence baseline for 2 seconds (excitation, 480 nm; emission, 530 nm), first add occurred by adding 20 μL of test compound to the cells, and the response was measured for 140 seconds. This is followed by second add; 10 μL (5X) of an EC_{20} concentration of Glu agonist was added to the cells, and the response of the cells was

measured for 125 seconds. Immediately, third add occurred by adding 12 ul (5X) of an EC_{80} concentration of Glu agonist and the response of the cells was measured for 90 seconds. Vehicle (0.6 % DMSO) in assay buffer was added to the control wells at the 1st add to ensure glutamate EC_{20}, EC_{80}, and EC_{Max} response in the absence of testing compounds. Calcium fluorescence was recorded as fold over basal fluorescence and raw data were normalized to the maximal response to Glu agonist (EC_{Max}). Compound-evoked decrease in calcium response in the presence of Glu EC_{80} agonist was determined as inhibition activity of negative allosteric modulator, and potency (IC_{50}) and maximum inhibition response (% Glu_{Min}) of compounds were determined using a four-parameter logistical equation using GraphPad Prism (La Jolla, CA) or the Dotmatics software platform (Woburn, MA) :

$$y = bottom + \frac{top - bottom}{1 + 10^{(LogEC50 - A)Hillslope}}$$

where *A* is the molar concentration of the compound; *bottom* and *top* denote the lower and upper plateaus of the concentration-response curve; HillSlope is the Hill coefficient that describes the steepness of the curve; and IC_{50} is the molar concentration of compound required to generate a response halfway between the *top* and *bottom*.

DMPK Methods

IV plasma-brain level determination (PBL) and pharmacokinetics (PK).

In-life phase

Compounds were formulated as a solution in ethanol, PEG400, and saline (1:4:5 v/v, respectively) at a concentration of 1 mg/mL and administered as a single 0.2 mg/kg IV dose (1 mL/kg) to male, Sprague Dawley rats (n = 1; 342 gram body weights) via injection into a surgically-implanted jugular vein catheter. Blood samples were collected serially from a surgically implanted carotid artery catheter in each animal over multiple post-administration time points (0.033, 0.117, 0.25, 0.5, 1, 2, 4, 7, and 24 hours) into chilled, K2EDTA anticoagulant-fortified tubes and immediately placed on wet ice. The blood samples were then centrifuged (1700 rcf, 5 minutes, 4 °C) in order to obtain plasma samples, which were stored at -80 °C until analysis by LC-MS/MS.

For determination of the brain over plasma ratio (K_p), compounds were formulated in 8% ethanol, 32% PEG400 and 60% DMSO (v/v/v) and administered as a single 0.2 mg/kg IV dose (1 mL/kg) to male, Sprague Dawley rats (n = 1; 316 gram body weights) via injection into a surgically-implanted jugular vein catheter. At 15 min post dosing, blood sample was collected serially (i.e., terminally) into chilled, K2EDTA anticoagulant-fortified tube and immediately placed on wet ice. The blood sample was then centrifuged

(1700 rcf, 5 minutes, 4 °C) to obtain plasma sample. At the same post-administration time point, whole brain sample was obtained by rapid dissection, rinsed with PBS, and immediately frozen in individual tissue collection box (dry ice). All brain and plasma samples were stored at -80 °C until analysis by LC-MS/MS.

Male Sprague-Dawley rats were used for discrete pharmacokinetic studies. Compounds in ethanol: PEG400: saline (1:4:5 v/v/v) were administered intra-venously in two rats at 1 mg/kg (IV PK). Two rats were also gavaged at 10 mg/kg with compounds in 10% Tween 80 in water (PO PK). Blood samples were collected from all mice at 8 different time points post-dose (0.25, 0.50, 1.0, 2.0, 4.0, 7.0 and 24 hr) and plasma was prepared as described above. The concentration of compounds in plasma was quantified by LC/MS/MS as described below.

The IV PK experiment was used to determine clearance, Volume of distribution and half-life. The PO experiments were used to determine exposure and oral bioavailability. PK parameters were calculated using Phoenix WinNonlin software (version 8.1).

Samples preparation for bioanalysis

Plasma samples from the in-life phase of the study were thawed at ambient temperature (benchtop), and then aliquots (20 µL per sample) were transferred to a 96-shallow-well (V-bottom) plate. Matrix-matched quality control (QC) samples and standard curves of **VU6031545** and **VU6024945** (1 mg/mL DMSO stock solution) were prepared in blank rat plasma (K2EDTA-treated) or blank brain homogenate via serial dilution and transferred (20 µL each) to the plate along with multiple blank plasma and brain homogenate samples. Acetonitrile (120 µL) containing IS (10 nM carbamazepine) was added to each well of the plate to precipitate protein. The plate was then centrifuged (4000 rcf, 5 minutes, ambient temperature), and resulting supernatants (60 µL each) were transferred to a new 96-shallow-well (V-bottom) plate containing an equal volume (60 µL per well) of water (Milli-Q purified). The plate was then sealed in preparation for LC-MS/MS analysis.

Preparation of brain samples was identical to that of plasma samples except for the following modifications. While thawing, brains were weighed (inside their collection boxes using a universal empty collection box tare weight) and then subjected to mechanical homogenization (Mini-BeadBeater™, BioSpec Products, Inc., Bartlesville, OK) in the presence of zirconia/silica beads (1.0 mm) and extraction buffer (isopropanol:water, 7:3, v/v; 3 mL per sample, corrected for post-quantitation). Homogenized brain samples were then centrifuged (4000 rcf, 5 minutes, ambient temperature), and 5 uL of the supernatant was diluted in 15 uL of blank plasma for quantification of the analyte. The plasma standard curve and QCs were used for compounds quantitation in brain.

Binding in plasma from rat and human.

Determination of compounds' fraction unbound (f_u) in plasma from rat and human was conducted *in vitro* via equilibrium dialysis using HTDialysis membrane plates. Dialysis membranes (four paired strips per HTD assay) were hydrated as described by the manufacturer and inserted into the HTD plate, which was assembled and prepared for sample addition by the dispensing of blank buffer (DPBS, 100 µL/well) into the 'top half' of each membrane-split well. Each compound was diluted into plasma from each species (5 µM final concentration), which was aliquoted in triplicate to the 'bottom half' of the prepared HTD plate wells. The HTD plate was sealed and incubated for 6 hours at 37 °C. Following incubation, each well (both top and bottom halves) were transferred (20 µL) to the corresponding wells of a 96-shallow-well (V-bottom) plate. The daughter plates were then matrix-matched (buffer side wells received equal volume of plasma, and plasma side wells received equal volume of buffer), and extraction solution (120 µL; acetonitrile containing 50 nM carbamazepine as IS) was added to all wells of both daughter plates to precipitate protein and extract test article. The plates were then sealed and centrifuged (3500 rcf) for 10 minutes at ambient temperature. Supernatant (60 µL) from each well of the daughter plates was then transferred to the corresponding wells of new daughter plates (96-shallow-well, V bottom) containing water (Milli-Q, 60 µL/well), and the plates were sealed in preparation for LC-MS/MS analysis (see below).

f_u was calculated as (analyte to IS MS peak area ratio from Trans-buffer side) / (analyte to IS MS peak area ratio from Cis-plasma side). Mean values for each species were calculated from 3 replicates.

Binding in brain homogenate from rat.

Determination of fraction unbound (f_u) in brain homogenate from rat was conducted using the same methodology and procedure than described for plasma protein binding assay with the following modifications: 1) a final compound concentration of 1 µM was used, 2) naïve rat brains were homogenized in DPBS (1:3 composition of brain: DPBS, w/w) using a Mini-Bead Beater™ machine in order to obtain brain homogenate.

The diluted fraction unbound (f_{u2}) in brain was calculated as (analyte to IS MS peak area ratio from Trans-buffer side) / (analyte to IS MS peak area ratio from Cis-brain homogenate side). Undiluted fraction unbound for the brain was calculated using the following equation:

$$f_u = \frac{1/4}{\left\{\left(\frac{1}{f_{u2}}\right) - 1\right\} + 1/4}$$

Mean values for each species were calculated from 3 replicates.

Intrinsic Clearance in Rat and Human Liver Microsomes

The *in vitro* intrinsic clearance (CL_{int}) was investigated in commercially obtained hepatic microsomes from rat and human donors using the substrate depletion (i.e., loss-of-parent vs. time, or $t_{1/2}$ method) approach with analyte detection via liquid chromatography-tandem mass spectrometry (LC-MS/MS). For each species, mean %parent remaining values at each time point were calculated from replicates raw data (analyte:IS peak area ratios) and used to determine *in vitro* $t_{1/2}$ and CL_{int}.

Experiments were carried out using a robot-assisted (TECAN model Evo 200). Compound was incubated (1 µM final concentration) in buffer (100 mM potassium phosphate pH 7.4 with 3 mM $MgCl_2$) containing hepatic microsomes (0.5 mg/mL final concentration) from multiple species, discretely, at 37 °C under constant orbital shaking. After 5 minutes (pre-incubation), reactions were initiated by addition of nicotinamide adenine dinucleotide phosphate (NADPH, 1 mM final concentration). At selected time intervals (0, 3, 7, 15, 25, and 45 minutes) post-addition of NADPH, aliquots (50 µL) were taken and placed into a 96-shallow-well plate containing ice cold acetonitrile (150 µL) with carbamazepine (IS, 50 nM). The plates were then centrifuged (3000 rcf at 4 °C) for 10 minutes. The supernatants were transferred to a new 96-shallow-well daughter plate and diluted (1:1 v/v) with water (Milli-Q filtered). The plates were then sealed in preparation for LC-MS/MS analysis (see below).

Raw LC-MS/MS peak area data generated from the assay samples were used to construct natural log-transformed %parent remaining vs. time plots (using t = 0 minute post-NADPH addition sample data as starting point set to 100%). *In vitro* compound half-life ($t_{1/2}$) values were obtained using the following equation:

$$t_{1/2} = \frac{Ln(2)}{k}$$

Where *k* is the slope from linear regression analysis of the natural log-transformed data (using means from all replicates at each time point). Resulting $t_{1/2}$ values were then used to calculate hepatic CL_{int} values according to the following equation and with the use of species-specific scale-up factors for liver weight (grams) per total body weight (kg):

$$CL_{int} = \frac{0.693}{in\ vitro\ t_{1/2}}\ x\ \frac{1\ mL\ incubation}{0.5\ mg\ microsomes}\ x\ \frac{45\ mg\ microsomes}{1\ gram\ liver}\ x\ \frac{\mathrm{a}\ gram\ liver}{kg\ body\ wt}$$

[a]Scale-up factors used are 45 (rat) and 20 (human).[1]

Predicted hepatic clearance (CL_{hep}) was calculated using the following equation:

$$CL_{hep} = \frac{Q_h * CL_{int}}{Q_h + CL_{int}}$$

Q_h represents hepatic blood flow (mL/min/kg): 21 for human, 70 for rat, and 90 for mouse.

Cytochrome P450 inhibition using Liver Microsomes.

The ability of compounds to inhibit cytochrome P450s (CYPs) was tested using human liver microsomes and a cocktail of substrates specific for CYP1A2, 2C9, 2D6 and 3A4. Compounds were incubated at 37C in presence of human liver microsomes (0.1 mg/ml) at 30, 10, 3, 1, 0.3 and 0.1 µM. A cocktail of substrate was also added for each CYP tested: phenacetin (10 µM, CYP1A2), diclofenac (5 µM, CYP2C9), dextromethorphan (5 µM, CYP2D6) and midazolam (2 µM, CYP3A4). HLM were also incubated with the control inhibitor miconazole at 3, 1, 0.3, 0.1, 0.03 and 0.01 µM. An aliquote of the reaction mixture was taken (0 hr time point) and the reaction was initiated by adding 1 mM of NADPH. After 8 minutes at 37C, the reaction was terminated by adding by adding 2 volumes of acetonitrile containing 50 nM of carbamazepine (internal standard).

The concentrations of CYP-specific metabolites generated at the end of the reaction were quantified by mass spectrometry using standard curves for each metabolite: acetaminophen (CYP1A2), 4-hydroxydiclofenac (CYP2C9), dextrophan (CYP2D6) and 1-hydroxymidazolam (CYP3A4). A concentration response curve for each metabolite was generated and IC_{50}s were calculated using Prism. The LC-MS/MS transitions for metabolite quantifications are as follows.

CYP450 Isozyme	Metabolite	MRM	DP	EP	CE	CXP
CYP1A2	Acetaminophen	M/Z 152.0→110.0	55	10	23	5
CYP2C9	4-Hydroxydiclofenac	M/Z 312.3→231.1	60	10	25	10
CYP2C19	4-Hydroxymephenytoin	M/Z 235.2→150.1	80	10	40	10
CYP2D6	Dextrorphan	M/Z 258.1→199.1	80	10	40	10
CYP3A4	1-Hydroxymidazolam	M/Z 342.3→203.1	80	10	40	10

Declustering potential (DP), entrance potential (EP), collision energy (CE), and collision cell exit potential (CXP)

LC-MS/MS Analysis

Prepared samples were injected (10 µL each) onto an AB Sciex Triple Quad 4500 mass spectrometer system with an Agilent 1260 Infinity II pump and autosampler. Mass spectrometer conditions are described in **Table S1.** Quantitation of compounds was performed via AB Sciex Multiquant software using the raw analyte:IS peak area ratios. The typical detection range was 0.5 ng/mL to ≥ 5,000 ng/mL utilizing a quadratic equation regression with 1/x2 weighting.

Correction for dilution of all brain samples (in extraction buffer and subsequently in blank plasma, as previously described) was performed post-quantitation. The corrections for dilution in extraction buffer employed correction factors specific to each brain weight (not shown).

Table S1. **LC-MS/MS Conditions***

Injection volume	10 µL	
Mobile phase A	0.5% Formic Acid in Water	
Mobile phase B	0.5% Formic Acid in Acetonitrile	
Flowrate	0.5 mL/min	
Gradient	Time	% Mobile Phase B
	0.0	5
	0.2	5
	0.8	95
	1.5	95
	1.7	5
	2.7	Stop
Column	Fortis C18 (50 x 3.0 mm, 3 µm)	
Data collection and analysis software/version	Analyst v. 1.7.1	
Ionization mode	Positive Electrospray	
Curtain gas (psi)	40	
GS1 (psi)	40	
GS2 (psi)	40	
Capillary voltage (V)	5500	
Source TurboIonSpray® temp. (°C)	500	

References

1) Lin J.H.; Chiba M.; Balani S.K; Chen I.W.; Kewi G.Y.; Vastag K.J.; Nishime J.A. Species differences in the pharmacokinetics and metabolism of indinavir, a potent human immunodeficiency virus protease inhibitor. *Drug Metab Dispos*. 1996, 24, 1111-1120.
